# Hospital Construction Competitions

**Published:** 1912-05-04

**Authors:** Edwin T. Hall

**Affiliations:** 54 Bedford Square, London, W.C.


### THE EDITOR'S LETTER-BOX.

### HOSPITAL CONSTRUCTION COMPETITIONS.

### EAST SUSSEX HOSPITAL.

*To the Editor of* The Hospital.

Sir,—I see in your issue of the 27th instant you have published my letter of the 15th instant, together with notes by "Nos. 1 and 2," and your own editorial note.

I am not satisfied with these notes.

Dealing first with your own comments:

I have purchased six copies of your paper, in which the word is "adviser," and I have one copy in which the word is "advisers."

In those with the word in the singular the full stop is immediately close to the "r," and in the other copy it is in a similar position in regard to the final "s."

There appears, therefore, no question of printer's error, but a deliberate alteration, and that two editions were published.

If, however, there were a printer's error, you are as responsible for that as for any other part of your paper, and I must press for a withdrawal and apology in so far as the matter relates to me.

With regard to your other observation, after stating that you see nothing to withdraw or for which to offer an apology, you go on to say that your contention regarding the rejection of plans on the score of cost "would not directly apply" to this Competition.

Having made this withdrawal, I still await the apology.

As to "F.R.I.B.A. No. 1," I would point out that in his note in your issue of the 27th instant he does not shelter himself under any explanation of printer's error, but confirms the offence, and goes on to make observations in regard to his mis-statement of the £35,000, but has not the grace to apologise even for this mis-statement, and I think your readers, on perusing his last note, will form their own judgment as to his judicial-mindedness and as to his powers of logical deduction.

As to the notes of "F.R.I.B.A. No. 2" regarding "cost," I think, in his own language, your "readers can judge" not only what the words of the Conditions imply, but what the words of his note imply.

With reference to the site, "No. 2" is good enough to say that I rely upon the answers to questions. I rely on the whole of the Conditions, of which the replies form only part. The original Condition "No. 4" says, "The plan furnished to each of the competitors shows the site, the levels, etc. . . . ." The plan in question is covered with levels showing the extreme variation of the surface. It speaks for itself to any technical man, and it would have been contrary to custom to make any such observation in the text of the Conditions as "No. 2" suggests.

I am, Sir, your obedient servant,

Edwin T. Hall.

54 Bedford Square, London, W.C.,
April 30, 1912.

[We willingly apologise to Mr. Hall for the printer's error so far as it may in any way relate to or affect himself.—Ed., The Hospital.]

